# Top Sodium Food Sources in the American Diet—Using National Health and Nutrition Examination Survey

**DOI:** 10.3390/nu15040831

**Published:** 2023-02-06

**Authors:** Mavra Ahmed, Alena (Praneet) Ng, Anthea Christoforou, Christine Mulligan, Mary R. L’Abbé

**Affiliations:** 1 Department of Nutritional Sciences, University of Toronto, Toronto, ON M5S 3E2, Canada; 2 Joannah and Brian Lawson Center for Child Nutrition, University of Toronto, Toronto, ON M5S 3E2, Canada

**Keywords:** sodium, dietary sources, dietary intake, NHANES, food category

## Abstract

Reducing population-level sodium intake can reduce hypertension, an important preventative strategy to lower the risk of cardiovascular diseases, the leading cause of death in the United States. Considering that most dietary sodium is derived from prepackaged foods, this study quantitatively estimates the proportion contribution and mean sodium intake from key food category contributors to total sodium intake in the US population. Data from the 2017–2018 National Health and Nutrition Examination Survey, which collected interviewer-administered 24 h dietary recalls from Americans (*n* = 7081), were analyzed. Based on the average proportion contributed, the top 15 sources of sodium were identified overall and by age/sex, poverty–income and race/ethnicity. More than 50% of US population-level dietary sodium intake was contributed by: pizza (5.3%); breads, rolls and buns (4.7%); cold cuts and cured meats (4.6%); soups (4.4%); burritos and tacos (4.3%); savoury snacks (4.1%); poultry (4.0%); cheese (3.1%); pasta mixed dishes (2.9%); burgers (2.5%); meat mixed dishes (2.5%); cookies, brownies and cakes (2.4%); bacon, frankfurters and sausages (2.4%); vegetables (2.2%); and chicken nuggets (1.5%), with the results remaining consistent among population subgroups. The results identified the top sources of sodium in the American population overall, as well as in key population subgroups, which can inform policies and programs aimed at reducing sodium intake.

## 1. Introduction

Nearly 90% of Americans consume sodium at levels which exceed amounts recommended by the 2015–2020 Dietary Guidelines for Americans [1]. Reducing population-level sodium intake can reduce blood pressure, an important preventative strategy to lower the risk of cardiovascular diseases, the leading causes of death in the United States [2]. It is well established that most dietary sodium is derived from prepackaged foods [3,4]. Efforts have been made to curtail population sodium intake through consumer education and food labelling campaigns, but have had minimal impact [5]. As a result, experts have recommended that sodium reduction in foods be a primary focus of public health strategies to achieve population-level sodium reduction [6]. To encourage the consumption of foods naturally low in sodium and effectively target the reduction of sodium levels in food, the identification of key food contributors to population-level sodium intake is crucial.

The National Health and Nutrition Examination Survey (NHANES) is a cross-sectional health and nutrition survey of non-institutionalized American residents conducted yearly in the United States. Previous analyses of 2015–2016 NHANES data elucidated that sandwiches; pizza; cured meat/poultry; mixed dishes (Mexican); and breads, rolls and tortillas were major sources of sodium in American’s diets [7,8,9]; however, updated data on the top food category contributors to dietary sodium intake are needed, given the continued prioritization of dietary health within US national dietary guidance [10] and the availability of more recent NHANES data [11]. Food categories identified by the Dietary Guidelines Advisory Committee (DGAC) to contribute meaningfully to dietary sodium have also emerged as important dietary contributors to other key nutrients and food groups [12]. For example, research has also shown that food groups such as mixed dishes, which have been identified as one of the top contributors to sodium intake, also accounted for half of total vegetable intake in children, therefore simultaneously contributing positively to the diet [13]. In contrast, other sodium contributing food groups such as savoury snacks could be the driving intakes of additional nutrients, for which there is guidance to discourage within the diet (e.g., saturated fat, calories) [9]. Therefore, understanding how the main dietary sources of sodium contribute to energy, nutrients and food group intakes will enhance the effective monitoring of national diet quality relative to recommended best practices. However, work is first needed to elucidate what the top contributing food categories to sodium intake are. Furthermore, evidence on how the contribution of various food categories to sodium intake impact different population subgroups (e.g., age and sex, race, or socioeconomic status (SES) groups) is needed to ensure that population-level dietary guidance, reformulation strategies and sodium-related nutrition policies are effective and equitable across population sub-groups.

The objective of this study was therefore to determine the top food category contributors of dietary sodium intake in the US population and provide quantitative estimates of sodium intake from these food categories, overall and by age/sex, race and household income level groups. These estimates were generated from the 2017–2018 National Health and Nutrition Examination Survey (NHANES) food and nutrient data.

## 2. Materials and Methods

### 2.1. Analytic Sample

This study was a cross-sectional analysis of nationally representative data from NHANES 2017–2018. NHANES participants completed an in-person Automated Multiple Pass Method (AMPM) 24 h dietary recall and general health examination in a Mobile Examination Center. A subsample of respondents completed a second 24 h dietary recall via telephone 3–10 days after the Center exam. Detailed descriptions of the survey design and the data collection procedures are available elsewhere [11]. In this study, analyses were conducted using the first day of dietary recall only. Previous NHANES studies have indicated a high reproducibility of the 24 h recall method between the in-person and telephone data collection. As indicated by Steinfeldt et al., there were no significant differences in energy intake between Day 1 and Day 2 and this was true across gender, ethnicity and income levels [14]. Additionally, the difference in energy intake was less than 4% for both males and females [14]. The United States Department of Agriculture (USDA) food composition database (Food and Nutrient Database for Dietary Studies (FNDDS)) was used to determine the amount of sodium (mg) in foods consumed by NHANES respondents as reported in their 24 h recall [15]. Full nutrient and dietary data were available for 7640 respondents in NHANES 2017–2018. Children under two years of age (*n* = 511) and lactating people (*n* = 48) were excluded from the analyses, leaving a total analytic sample of 7081. For analyses across household income subgroups, a further 1753 were removed due to missing income data.

### 2.2. Food Categorization

The FNDDS database were used to define food categories within NHANES 2017–2018 dietary intake data [15]. For the present analyses, FNDDS food codes were aggregated into 87 categories, adapted from the What We Eat In America (WWEIA) food categories (Supplementary Table S1 [16]), and consistent with previous analyses of the top food sources of sodium intake in the American diet [17]. All individually consumed foods were then assigned to one of these mutually exclusive categories (e.g., tortillas, cooked cereals) [11], representative of items ‘as consumed’ (e.g., cheese sandwich, rather than bread and cheese separately).

### 2.3. Statistical Analyses

The percentage contribution of each of the 87 food categories to total daily dietary sodium intake was calculated and ranked. A population ratio approach was used to rank the 87 food categories in descending order by their contribution to overall population total daily dietary sodium, shown in Supplementary Table S1. Briefly, the sodium intake provided by each food category was summed across all individuals in the sample and divided by the sum of total daily intake of sodium for all individuals in the sample. In contrast to calculating ratios for each individual and averaging across the sample, the population ratio approach better reflects usual intake [18] and is consistent with methods used previously [17,19,20].

The percentage contribution of the top 15 food categories to total daily dietary sodium intake was then calculated. The weighted mean sodium contribution (mg/day ± standard error) of the top 15 categories was also estimated for consumers of each individual food category, and per capita. All weighted means were unadjusted for salt that may have been added during food preparation [9].

To inform equitable approaches to support healthy eating for all Americans, analyses were completed for the overall population, and by Dietary Reference Intake (DRI) age/sex groups [21], race and household income level groups. Racial groups were defined as White, non-Hispanic Black, Hispanic, Asian and Other as per the self-reported data collected in NHANES. Household income groups were defined using the Poverty Income Ratio (PIR), which represents the ratio of household income to the federal poverty threshold, adjusted for inflation and family size and composition [22]. Household income level groups for these analyses were determined using PIR cut-points based on eligibility criteria for food assistance programs: “lowest” (PIR ≤ 180% of the threshold), “middle” (180% < PIR ≤ 350%) and “highest” (PIR >350%) [22]. This study did not adjust for misreporting, in accordance with previous analyses conducted using NHANES datasets [23,24], as excluding participants on the basis of presumed misreporting (e.g., using the Goldberg equation) can potentially introduce bias by affecting the representativeness of the sample [19]. There were no significant differences in PIR across ethnicities.

Descriptive statistics were performed in SAS Version 9.4 (SAS Institute, Inc.; Cary, NC, USA) using the first day of 24 h dietary recall. All results are presented as percentage contribution (%) and means (mg/day) ± standard error. Due to the complex sampling design of NHANES, robust standard errors were computed using the Taylor series linearization method [25]. All percentages and means were weighted using sampling survey weights provided in NHANES 2017–2018 to ensure population-level estimates.

## 3. Results

**Figure 1:**
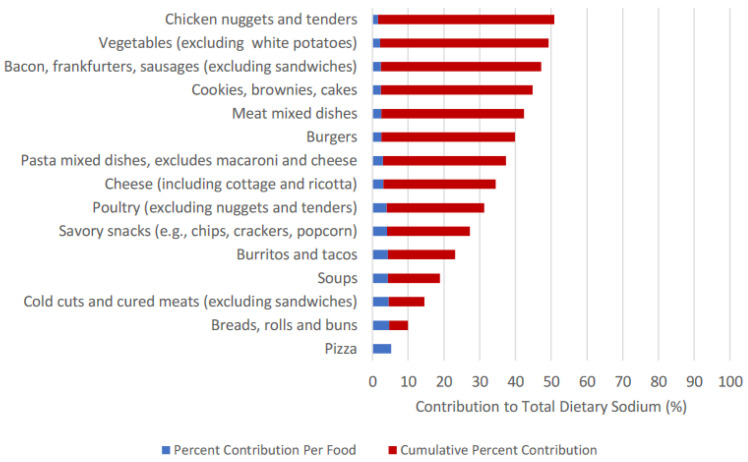
Percent Contribution and Cumulative Percent Contribution of Top 15 Food Categories Contributing to Sodium Intake.

In sum, the top 15 food categories accounted for 50.83% to total dietary sodium intake (Table 1, Figure 1): pizza (5.3%); breads, rolls and buns (4.7%); cold cuts and cured meats (4.6%); soups (4.4%); burritos and tacos (4.3%); savoury snacks (4.1%); poultry (4.0%); cheese (3.1%); pasta mixed dishes (2.9%); burgers (2.5%); meat mixed dishes (2.5%); cookies, brownies and cakes (2.4%); bacon, frankfurters, sausages (2.4%); vegetables (2.2%); and chicken nuggets (1.5%) (Figure 1).

Within DRI age/sex groups, the percentage sodium contribution of these categories ranged from 49.1% in 51–70-year-old males to 56.3% in 9–13-year-old males. These 15 food categories had the highest sodium contribution in the ‘Other’ race group (53.0%), but this was also relatively consistent across all race groups, ranging from 44.1% to 51.6%. The percentage contribution was also consistent across household income level groups, ranging from 50.7% in the “lowest” household income, to 51.6% in the “highest” group. The exact ranking of the percentage contribution of each individual food category varied across all analyzed subgroups (Table 1).

Table 2 displays the mean sodium intakes (mg/day ± SE (*n*)) related to the top 15 food categories overall and by subgroups, for consumers of that specific food group. Mean sodium intakes varied by food category and by the analyzed subgroups. Overall, burritos and tacos contributed the highest amount of daily sodium, with consumers (*n* = 618) consuming 1656 ± 49 mg/day on average from this food category alone. Burritos and tacos had the highest mean intake level across consumers from all household incomes and race groups and among most DRI age/sex groups. Chicken nuggets and tenders had the lowest mean daily intake out of the top 15 categories, with consumers (*n* = 2866) consuming, on average, 167 ± 6.8 mg/day of sodium from these product types. This was also consistent across most analysed subgroups. Mean sodium intakes per capita (i.e., including consumers and non-consumers of that food category) are presented in Table 3. On a per capita basis, pizza had the highest mean intake at 158 ± 5.4 mg/day of sodium and the lowest mean daily intake was from vegetable at 52 ± 4 mg/day of sodium. Although this was consistent across the majority of the subgroups, the mean daily intake of sodium from cold cuts and cured meats was highest among some subgroups (e.g., White).

## 4. Discussion

This study found that the top 15 food category contributors to dietary sodium represent just over 50% of total dietary sodium intake for American adults, with pizza, breads, cold cuts, soups and burritos being the top five contributors. Our findings were consistent across the population subgroups that were investigated with some small variations. These estimates are in line with previous research using NHANES and Canadian data which has shown that the top ten sources of sodium intake represented approximately 40–60% (in different populations/years) of the total dietary contribution of sodium [9,19,26].

The top dietary sources of sodium reported in this study are also in close agreement with those reported by the Centers for Disease Control and Prevention using data from NHANES 2013–2014 [8]. The only notable difference was that “eggs and omelettes” was indicated as one of the top 10 dietary sources in the earlier CDC study, whereas this category only fell within the top 20 food categories (i.e., just beyond the top 15) in the present analyses. However, our analyses found that food categories that fell just beyond the top 15 categories, such as “eggs and omelets”, had very similar percent sodium contributions as vegetables (ranked 15, 2.2%), ranging from 2% to 1.8%. The results of this study are also comparable to analyses reporting on Canadian intake data which also highlighted that meats, breads, pasta, pizza and soups are top contributors to Canadian dietary sodium intake [19,26].

It is worth noting that the top 15 contributing categories to sodium intake also consistently ranked in the top 15 categories across age/sex, race/ethnicity and household income groups, although the exact ranking of the food category may have varied (e.g., soups ranked first in the Asian group, whereas soups ranked eleventh in the Black group). Moreover, there were only a few additional food categories that ranked in the top 15 for specific age/sex, race/ethnicity and household income groups (e.g., enchiladas and fajitas ranked ninth for the Hispanic group). This variability is likely a function of broader social, economic and environmental factors that play a role in food choices and dietary habits relating to specific food groups (e.g., pizza is popular among teens), social desirability bias and other social pressures, health concerns, education/health literacy, the incorporation of culturally diverse or traditional foods and food availability and accessibility (e.g., food prices) [27].

There are several reasons why a food category may have been found to be a top contributor to sodium intake. For example, although breads and vegetables were found to be top contributors, this was likely due to the amount of these food categories consumed, rather than their high concentration of sodium. On the other hand, cold cuts and soups are consumed in smaller amounts, but contain high concentrations of sodium, meaning that they are still top contributors to overall dietary sodium intake. There are also food categories such as frozen dinners which are known to be very sodium-dense, but are only consumed by a small portion of the population [26]. For those individuals who consume these food types, these food types likely contribute toward a significant proportion of their dietary sodium from a single meal; however, analyses at the population level will not capture such categories as top contributors [26], and thus, we also present the results of consumers only in Table 2. Therefore, while identifying the top population-level sodium sources to inform dietary recommendations is integral to improving dietary health on a large scale, for at-risk or vulnerable individuals, it is also important to consider dietary modifications at the individual level.

The majority of Americans consume sodium at levels which exceed amounts recommended by the 2015–2020 Dietary Guidelines for Americans [1] and methods such as 24 h urine collection are considered the gold standard in estimating usual sodium intake for individuals. Previous NHANES studies showed comparable estimates of sodium intake with urinary sodium excretion [28,29,30]. Different strategies have been adopted by several countries to tackle increasing sodium intake and reduce the level of sodium in the food supply. The Food and Drug Administration (FDA) is taking an iterative approach that includes the establishment of voluntary sodium targets for the industry, monitoring and evaluating progress and engaging with stakeholders. The guidance includes measurable voluntary short-term targets (2.5 years) for sodium reduction in commercially processed, packaged and prepared foods to reduce excess population sodium intake [31]. The FDA estimates a 12% reduction in sodium intakes if the sodium reduction targets are fully implemented by the industry [31]. Setting sodium reduction targets for packaged foods has been shown to encourage reformulation with lower sodium content in processed foods and reduce the population sodium intake in some countries [32,33]. For example, in 2006, the United Kingdom established progressively lower voluntary sodium targets for over 80 food categories, provided clear timetables to encourage ongoing food reformulation and monitored the progress of sodium reduction by the industry. As a result, the population sodium intake measured by 24 h urinary excretion declined by 10% between June 2005 (mean {95% CI, 3304 (69–6539)} mg/d) and 2011 (2989 {494–5484} mg/d). About 60% of the reductions occurred between June 2005 and September 2008 [33]. On the other hand, results examining food industry progress in reducing the sodium content of packaged foods in Canada from 2010 to 2016 indicated that the proportion of foods meeting at least one of the three phases of the sodium reduction benchmark targets slightly increased (51.4% to 58.2%) and the proportion exceeding the maximum benchmark levels decreased (25.2% to 20.8%) [34].

The present research contributes important information pertaining to the food categories that would be amenable to reformulation and have significant impact on Americans’ diets. These include various meat products (e.g., red meat and poultry dishes, burgers, and processed meats), vegetable dishes (e.g., mixed vegetable dishes), pasta and rice dishes, pizza, soups, breads and other baked goods (e.g., cookies and muffins). Future analyses examining the sodium contributions of more disaggregating food categories (e.g., food subcategories, such as breaded vs. plain chicken) could provide additional insights to inform reformulation efforts. The implementation of additional complimentary nutrition policies and programs (e.g., front-of-package labelling, marketing restrictions, nutrition education) can further facilitate and encourage food reformulation and dietary shifts away from top sodium contributing categories, either at a population level, or tailored to specific population subgroups.

This study presents an important update to the understanding of the food categories that are the most significant contributors of dietary sodium intake among American adults. This work was strengthened by its analysis of NHANES data, using robust methodology. For instance, the NCI method was used to estimate mean usual intakes [35] and the population ratio approach, which has been found to better reflect usual intakes, was used, rather than calculating ratios for each individual and averaging them across the sample [18,20,36]. Importantly, this study also examined sodium contributions among key subgroups (i.e., age/sex, race/ethnicity and income), providing a more detailed and granular analysis of sodium contributions to the diet.

However, there are some limitations to consider, many of which are inherent to the use of NHANES data. Our findings cannot be generalized to subpopulations not included in the NHANES, such as people in supervised care or custody in institutional settings, active-duty military personnel and U.S. citizens residing outside of the 50 states and the District of Columbia [11]. Additionally, while NHANES uses the AMPM 24 hr recalls, which have been shown to be less susceptible to bias than other traditional dietary assessment methods (e.g., food frequency questionnaire) [37,38,39,40,41,42], dietary intake is still well-known to be reported with bias due to errors in recall (forgetting foods or beverages) [38,43], the misestimation of portion sizes [44,45,46] and social desirability biases that might lead to the misreporting of particular foods [47]. As such, due to the differential misreporting of certain food types [38,43], it is possible that the contributions of food and beverage categories commonly perceived as unhealthy (e.g., sugary beverages, cookies, cakes and burgers) may be underestimated, whereas the contributions of categories perceived as healthy (e.g., fresh and frozen fruits and vegetables, and milk) may be overestimated. Differences in misreporting also exist across individuals, with implications for comparisons across groups. For example, misreporting resulting in the underestimation of energy intake has been shown to be associated with being female, older or Black, and having body dissatisfaction, higher household income or higher body mass [38,40,48], while overreporting can be associated with being male, younger, a smoker or underweight, and having lower household income [48].

Finally, although the food categories used in this study were informed by the NHANES What We Eat In America food categories, there is still some heterogeneity across categories (e.g., “mixed dishes” contains meat-only dishes, but also meat dishes that contain vegetables) which may have diluted the overall potential contribution of that particular food category to sodium intakes in the diet. Further research should aim to examine sodium contributions at a more detailed sub-category level. Moreover, to continue to improve our understanding of food category contributions to sodium intake, future research in this area should aim to examine sodium intakes across additional socioeconomic and demographic variables.

## 5. Conclusions

Overall, these analyses found that the top 15 contributing categories to dietary sodium intake accounted for just over 50% of the total dietary sodium intake in American adults. This study provides an updated understanding of the top sources of sodium intake in the American population overall, as well as in key population subgroups. Importantly, the results of this work can be used to inform population-level and subgroup-specific policies and programs aimed at reducing sodium intake to support healthy eating and reduce chronic disease risk in the United States.

## Figures and Tables

**Table 1:** Contribution to total daily sodium by food category (%), NHANES 2017–2018 ^1^ for the total sample and by sub-groups.

		Food Categories ^2^															
Overall Food Category Rank		1	2	3	4	5	6	7	8	9	10	11	12	13	14	15	Total % Contribution of Top 15 Categories
	*n*	Pizza	Breads, Rolls and Buns	Cold Cuts and Cured Meats (Excluding Sandwiches)	Soups	Burritos and Tacos	Savory Snacks (e.g., Chips, Crackers, Popcorn)	Poultry (Excluding Nuggets and Tenders)	Cheese (Including Cottage and Ricotta)	Pasta Mixed Dishes, Excludes Macaroni and Cheese	Burgers	Meat Mixed Dishes	Cookies, Brownies and Cakes	Bacon, Frankfurters and Sausages (Excluding Sandwiches)	Vegetables (Excluding White Potatoes)	Chicken Nuggets and Tenders	
Total sample	7081	5.26%	4.66%	4.59%	4.34%	4.29%	4.14%	4.03%	3.11%	2.90%	2.52%	2.47%	2.44%	2.38%	2.16%	1.54%	50.83%
**Household income ^3^**																	
Low	2029	5.56%	4.08%	3.37%	4.75%	5.01%	3.79%	4.44%	3.05%	3.32%	3.01%	1.77%	2.39%	2.24%	1.58%	2.37%	50.73%
Middle	2545	5.77%	4.59%	5.39%	4.02%	4.25%	4.64%	3.48%	3.05%	2.43%	1.64%	2.81%	2.68%	2.40%	2.16%	1.47%	50.78%
High	754	5.41%	5.11%	5.41%	3.39%	3.80%	3.57%	4.01%	3.11%	3.52%	2.25%	2.86%	2.43%	2.98%	2.25%	1.49%	51.59%
**Race/Ethnicity**																	
White	2479	5.23%	4.91%	5.41%	3.71%	3.20%	4.55%	3.12%	3.53%	2.86%	2.68%	3.06%	2.42%	2.72%	2.17%	1.56%	51.13%
Black	1646	5.05%	3.64%	3.93%	2.68%	3.33%	4.69%	7.71%	2.64%	3.45%	2.89%	1.41%	2.69%	2.66%	2.28%	2.57%	51.62%
Hispanic	1627	5.77%	3.92%	3.14%	6.33%	9.38%	3.24%	4.39%	2.58%	2.19%	2.21%	1.21%	2.18%	1.65%	1.79%	1.01%	50.99%
Asian	853	4.03%	6.26%	2.18%	9.25%	2.35%	2.11%	4.72%	1.22%	1.62%	1.08%	1.93%	2.06%	1.05%	3.31%	0.90%	44.07%
“Other”	476	5.68%	4.74%	4.09%	3.32%	3.91%	3.69%	4.44%	3.24%	5.75%	2.48%	2.87%	3.14%	2.01%	2.00%	1.67%	53.03%
**Dietary Reference Intakes Age–Sex Groupings**																	
>1 y	299	5.28%	3.69%	2.62%	2.89%	3.82%	7.19%	2.73%	5.20%	2.78%	0.95%	1.23%	2.89%	4.22%	1.71%	5.07%	52.27%
4–8 y	623	6.89%	4.36%	4.85%	2.77%	2.92%	6.23%	3.21%	3.76%	2.85%	1.89%	1.55%	3.58%	2.10%	1.18%	3.30%	51.44%
9–13 y males	328	9.61%	4.50%	3.68%	2.26%	6.38%	8.68%	3.57%	2.76%	3.08%	1.87%	0.96%	2.64%	2.51%	0.94%	2.85%	56.29%
9–13 y females	353	8.86%	3.99%	3.05%	3.68%	2.10%	6.38%	3.57%	3.24%	3.82%	1.57%	1.78%	3.65%	1.47%	1.40%	2.88%	51.44%
14–18 y males	341	9.32%	4.42%	6.27%	3.90%	3.62%	5.18%	4.63%	2.34%	3.00%	3.96%	1.26%	2.01%	1.36%	0.65%	3.00%	54.92%
14–18 y females	323	7.23%	5.19%	3.74%	3.50%	4.02%	5.93%	7.28%	2.73%	2.61%	2.07%	0.99%	2.63%	1.23%	1.51%	3.08%	53.74%
19–30 y males	422	6.96%	3.16%	5.98%	1.74%	7.41%	3.24%	4.05%	2.07%	2.78%	3.42%	2.38%	0.71%	2.00%	1.31%	2.39%	49.60%
19–30 y females	436	5.42%	3.41%	2.68%	3.08%	5.74%	3.99%	4.06%	3.43%	3.59%	2.03%	2.73%	1.82%	2.88%	2.69%	2.26%	49.81%
31–50 y males	677	5.88%	4.35%	4.01%	5.25%	6.33%	3.01%	5.34%	3.00%	2.06%	3.48%	1.19%	2.08%	2.52%	1.83%	0.85%	51.18%
31–50 y females	744	4.76%	4.86%	3.47%	4.54%	5.02%	4.02%	4.02%	3.36%	2.37%	2.24%	1.99%	2.52%	3.07%	3.48%	0.98%	50.70%
51–70 y males	876	3.71%	5.29%	5.72%	4.19%	2.79%	3.92%	3.67%	3.24%	3.54%	2.42%	2.88%	2.80%	2.44%	1.96%	0.50%	49.07%
51–70 y females	900	3.23%	5.65%	3.96%	6.28%	2.18%	3.63%	3.34%	3.59%	3.24%	2.32%	4.62%	2.60%	1.83%	3.18%	0.94%	50.59%
>70 y males	390	2.43%	5.87%	7.55%	6.99%	0.84%	2.52%	2.48%	2.04%	2.36%	1.96%	4.69%	3.80%	2.93%	2.10%	0.83%	49.39%
>70 y females	369	1.06%	6.29%	5.55%	6.96%	1.54%	4.60%	3.13%	3.89%	3.43%	1.19%	4.03%	3.61%	2.90%	3.80%	0.62%	52.60%

^1^ The population percentage contribution (%) of sodium from each food category to total daily dietary sodium intake was calculated as the sum of the amount of sodium consumed from each specific food category for all participants in the designated subgroup, divided by the total daily sodium intake for all participants. All estimates were generated from the first 24 h dietary recall and survey weighted to be representative at the population level. ^2^ This analysis used 87 food categories, which were adapted from What We Eat In America (https://www.cdc.gov/nchs/nhanes/wweia.htm) (accessed on 5 December 2020). Food categories are ranked in descending order by population percentage contribution among the total sample aged >1 y, excluding pregnant and lactating women (*n* = 7081). ^3^ Poverty-to-income ratio (PIR) was used to define household income. A PIR ≤ 180% was considered “low” income, a 180 < PIR ≤ 350 was considered “middle” income and a PIR > 350 was considered “high” income.

**Table 2:** Mean sodium intakes [mg/day ± SE (*n*)] by top sodium-contributing categories, NHANES 2017–2018, for the total sample and by sub-groups, consumers only ^1^.

Food Categories ^2^															
	Pizza	Breads, Rolls and Buns	Cold cuts and Cured Meats (Excluding Sandwiches)	Soups	Burritos and Tacos	Savory Snacks (e.g., Chips, Crackers, Popcorn)	Poultry (Excluding Nuggets and Tenders)	Cheese (Including Cottage and Ricotta)	Pasta Mixed Dishes, Excludes Macaroni and Cheese	Burgers	Meat Mixed Dishes	Cookies, Brownies and Cakes	Bacon, Frankfurters and Sausages (Excluding Sandwiches)	Vegetables (Excluding White Potatoes)	Chicken Nuggets and Tenders
Total sample	1388 ± 49 (930)	385 ± 9.5 (2779)	901 ± 36 (1075)	1238 ± 45 (920)	1656 ± 49 (618)	303 ± 9.0 (3186)	678 ± 27 (1502)	315 ± 11 (1986)	931 ± 39 (703)	792 ± 19 (702)	980 ± 58 (513)	264 ± 10 (2225)	552 ± 34 (976)	167 ± 6.8 (2866)	742 ± 36 (572)
**Household income ^3^**															
Low	1443 ± 75 (287)	361 ± 13 (718)	691 ± 39 (298)	1281 ± 118 (268)	1716 ± 88 (191)	292 ± 12 (859)	735 ± 60 (423)	325 ± 17 (547)	1020 ± 78 (199)	785 ± 33 (231)	990 ± 168 (107)	293 ± 28 (601)	591 ± 59 (257)	155 ± 10 (687)	870 ± 85 (178)
Middle	1396 ± 83 (344)	381 ± 15 (1012)	947 ± 62 (423)	1244 ± 60 (312)	1699 ± 83 (221)	332 ± 16 (1175)	654 ± 42 (500)	323 ± 18 (686)	888 ± 54 (255)	828 ± 33 (250)	1045 ± 93 (220)	293 ± 20 (806)	582 ± 69 (359)	172 ± 11 (975)	717 ± 52 (200)
High	1434 ± 118 (85)	390 ± 24 (318)	1084 ± 135 (110)	1134 ± 123 (100)	1523 ± 128 (50)	283 ± 23 (353)	766 ± 105 (154)	318 ± 43 (225)	1071 ± 108 (79)	764 ± 57 (78)	879 ± 97 (66)	245 ± 19 (266)	666 ± 132 (105)	159 ± 20 (360)	781 ± 123 (71)
**Race/Ethnicity**															
White	1331 ± 69 (334)	386 ± 14 (1093)	941 ± 48 (491)	1286 ± 78 (225)	1475 ± 68 (163)	331 ± 13 (1214)	622 ± 42 (353)	317 ± 16 (872)	883 ± 51 (269)	785 ± 25 (292)	1063 ± 81 (237)	253 ± 14 (820)	560 ± 49 (393)	160 ± 10 (987)	731 ± 55 (229)
Black	1480 ± 85 (216)	359 ± 13 (541)	888 ± 88 (235)	1080 ± 67 (146)	1779 ± 131 (95)	315 ± 13 (814)	866 ± 41 (469)	346 ± 23 (383)	1039 ± 77 (169)	864 ± 47 (174)	837 ± 90 (86)	290 ± 24 (525)	507 ± 21 (311)	200 ± 12 (606)	858 ± 67 (169)
Hispanic	1548 ± 108 (207)	368 ± 16 (569)	761 ± 58 (214)	1186 ± 66 (290)	1943 ± 93 (275)	285 ± 16 (628)	623 ± 60 (373)	292 ± 20 (460)	896 ± 80 (131)	762 ± 32 (151)	773 ± 69 (72)	260 ± 16 (466)	578 ± 68 (143)	145 ± 9.3 (654)	656 ± 55 (88)
Asian	1302 ± 125 (100)	469 ± 25 (403)	986 ± 111 (58)	1328 ± 95 (200)	1557 ± 135 (43)	196 ± 13 (307)	656 ± 69 (218)	284 ± 30 (128)	817 ± 72 (69)	840 ± 127 (39)	710 ± 89 (72)	239 ± 15 (273)	536 ± 69 (64)	214 ± 15 (456)	692 ± 90 (40)
“Other”	1426 ± 125 (73)	384 ± 47 (173)	779 ± 172 (77)	1088 ± 204 (59)	1476 ± 143 (42)	308 ± 29 (223)	861 ± 111 (89)	317 ± 34 (143)	1233 ± 202 (65)	792 ± 80 (46)	891 ± 128 (46)	381 ± 71 (141)	517 ± 84 (65)	193 ± 31 (163)	752 ± 67 (46)
**Dietary Reference Intakes Age–Sex Groupings**															
>1 y	640 ± 94 (51)	199 ± 21 (94)	387 ± 58 (40)	519 ± 61 (37)	927 ± 205 (16)	236 ± 24 (186)	277 ± 25 (64)	228 ± 26 (106)	390 ± 56 (37)	401 ± 63 (18)	438 ± 52 (15)	158 ± 13 (109)	494 ± 79 (54)	89 ± 14 (114)	441 ± 33 (65)
4–8 y	951 ± 80 (136)	288 ± 16 (236)	626 ± 76 (107)	908 ± 91 (57)	984 ± 141 (45)	280 ± 18 (365)	403 ± 29 (120)	272 ± 18 (189)	565 ± 60 (76)	522 ± 37 (60)	667 ± 164 (36)	213 ± 15 (248)	408 ± 41 (83)	98 ± 11 (188)	522 ± 40 (110)
9–13 y males	1185 ± 124 (86)	337 ± 21 (130)	557 ± 61 (61)	885 ± 117 (38)	1523 ± 195 (39)	494 ± 58 (175)	752 ± 173 (63)	287 ± 38 (79)	981 ± 160 (33)	642 ± 46 (30)	943 ± 212 (11)	242 ± 34 (116)	462 ± 39 (47)	96 ± 18 (87)	736 ± 147 (31)
9–13 y females	982 ± 121 (88)	346 ± 26 (105)	646 ± 84 (39)	1010 ± 94 (44)	1285 ± 125 (28)	329 ± 34 (205)	668 ± 83 (52)	309 ± 39 (91)	785 ± 124 (47)	656 ± 58 (35)	622 ± 102 (17)	231 ± 25 (146)	382 ± 45 (40)	127 ± 16 (112)	611 ± 58 (50)
14–18 y males	2008 ± 190 (71)	507 ± 61 (113)	1237 ± 190 (59)	1295 ± 275 (41)	1713 ± 158 (32)	412 ± 41 (164)	1128 ± 201 (53)	341 ± 41 (88)	1155 ± 150 (42)	865 ± 71 (58)	977 ± 203 (18)	298 ± 55 (104)	687 ± 79 (28)	125 ± 34 (72)	846 ± 93 (42)
14–18 y females	1233 ± 161 (54)	428 ± 33 (100)	792 ± 83 (37)	995 ± 91 (37)	1716 ± 306 (28)	359 ± 34 (160)	771 ± 89 (75)	320 ± 47 (72)	846 ± 147 (27)	618 ± 59 (34)	1252 ± 336 (10)	225 ± 30 (101)	413 ± 109 (26)	113 ± 18 (102)	1085 ± 165 (29)
19–30 y males	1932 ± 247 (67)	419 ± 34 (119)	1401 ± 176 (60)	1019 ± 112 (30)	2120 ± 138 (65)	320 ± 29 (158)	967 ± 157 (84)	306 ± 33 (109)	1265 ± 119 (46)	929 ± 57 (65)	959 ± 151 (34)	226 ± 25 (80)	555 ± 98 (48)	167 ± 22 (127)	1159 ± 198 (41)
19–30 y females	1216 ± 136 (62)	345 ± 25 (138)	718 ± 129 (51)	1253 ± 137 (43)	1430 ± 110 (53)	315 ± 35 (182)	547 ± 56 (111)	325 ± 33 (133)	1044 ± 117 (51)	731 ± 45 (42)	806 ± 76 (41)	205 ± 37 (123)	748 ± 283 (57)	192 ± 18 (188)	864 ± 84 (40)
31–50 y males	1734 ± 176 (83)	465 ± 36 (267)	1093 ± 103 (103)	1599 ± 174 (90)	1933 ± 147 (84)	327 ± 24 (248)	937 ± 101 (169)	404 ± 50 (184)	1455 ± 240 (47)	922 ± 66 (91)	747 ± 94 (61)	351 ± 44 (177)	694 ± 96 (94)	197 ± 23 (253)	1039 ± 248 (30)
31–50 y females	1219 ± 122 (79)	411 ± 31 (265)	787 ± 84 (75)	1229 ± 135 (107)	1719 ± 135 (73)	270 ± 31 (304)	526 ± 58 (196)	271 ± 19 (235)	667 ± 76 (69)	762 ± 41 (59)	884 ± 166 (45)	264 ± 32 (200)	619 ± 151 (96)	194 ± 25 (364)	652 ± 76 (39)
51–70 y males	1720 ± 262 (65)	412 ± 25 (420)	1004 ± 122 (170)	1310 ± 111 (132)	1615 ± 130 (66)	352 ± 37 (342)	769 ± 76 (205)	363 ± 41 (233)	1168 ± 126 (74)	828 ± 58 (93)	1316 ± 222 (72)	333 ± 36 (277)	541 ± 51 (147)	175 ± 21 (392)	643 ± 107 (25)
51–70 y females	1262 ± 103 (59)	359 ± 32 (387)	736 ± 45 (121)	1227 ± 110 (147)	1338 ± 133 (14)	219 ± 18 (363)	520 ± 35 (185)	303 ± 37 (257)	768 ± 62 (86)	729 ± 47 (62)	1203 ± 180 (71)	271 ± 35 (252)	395 ± 35 (115)	167 ± 17 (484)	656 ± 132 (44)
>70 y males	1455 ± 103 (16)	391 ± 23 (210)	1206 ± 178 (88)	1484 ± 183 (57)	1785 ± 190 (12)	214 ± 20 (164)	691 ± 83 (55)	274 ± 26 (103)	928 ± 122 (32)	753 ± 57 (34)	1213 ± 220 (48)	292 ± 25 (145)	561 ± 84 (79)	139 ± 18 (175)	656 ± 158 (14)
>70 y females	1054 ± 129 (13)	324 ± 17 (195)	665 ± 61 (64)	1076 ± 115 (60)	1146 ± 108 (13)	262 ± 34 (170)	495 ± 56 (70)	283 ± 30 (107)	957 ± 156 (36)	666 ± 72 (21)	854 ± 123 (34)	213 ± 21 (147)	500 ± 93 (62)	172 ± 15 (208)	555 ± 118 (12)

^1^ Means ± SE were generated using a PROC SURVEYMEANS procedure in SAS 9.4. All estimates were generated from one 24 h dietary recall and survey weighted to be representative at the population level. Standard errors were computed using the Taylor series linearization method to account for the complex survey design of NHANES. ^2^ This analysis used 87 food categories, which were adapted from What We Eat In America (https://www.cdc.gov/nchs/nhanes/wweia.htm) (accessed on 5 December 2020). Food categories are ranked in descending order by population proportion among the total sample aged >1 y, excluding pregnant and lactating women (*n* = 7081). ^3^ Poverty-to-income ratio (PIR) was used to define household income. PIR ≤ 180% was considered “low” income, 180 < PIR ≤ 350 was considered “middle” income and PIR > 350 was considered “high” income.

**Table 3:** Mean sodium intakes [mg/day ± SE (*n*)] by top sodium-contributing categories, NHANES 2017–2018, for the total sample and by sub-groups, per capita (full sample) ^1^.

	Food Categories ^2^															
	*n*	Pizza	Breads, Rolls and Buns	Cold Cuts and Cured Meats (Excluding Sandwiches)	Soups	Burritos and Tacos	Savory Snacks (e.g., Chips, Crackers, Popcorn)	Poultry (Excluding Nuggets and Tenders)	Cheese (Including Cottage and Ricotta)	Pasta Mixed Dishes, Excludes Macaroni and Cheese	Burgers	Meat Mixed Dishes	Cookies, Brownies and Cakes	Bacon, Frankfurters and Sausages (Excluding Sandwiches)	Vegetables (Excluding White Potatoes)	Chicken Nuggets and Tenders
Total sample	7081	178 ± 10.4	157.6 ± 5.39	155 ± 9.25	147 ± 9.02	145 ± 9.42	140 ± 5.09	136 ± 7.05	105 ± 4.95	98.2 ± 6.69	85.3 ± 5.08	83.6 ± 8.11	82.3 ± 3.86	80.8 ± 6.40	52.3 ± 3.94	73.4 ± 3.41
**Household income ^3^**																
Low	2029	180 ± 18.4	132 ± 6.96	109 ± 10.5	154 ± 19.5	162 ± 16.4	122 ± 6.47	143 ± 14.7	98.6 ± 7.64	107 ± 13.7	97.2 ± 8.55	57.3 ± 12.1	77.2 ± 8.51	72.4 ± 9.64	76.5 ± 11.2	51.1 ± 3.75
Middle	2545	194 ± 17.8	155 ± 8.26	181 ± 16.9	135 ± 12.3	143 ± 15.6	156 ± 9.59	117 ± 10.3	103 ± 7.54	81.9 ± 8.30	88.9 ± 9.13	94.7 ± 14.6	90.4 ± 7.90	80.8 ± 11.4	49.6 ± 5.67	72.8 ± 5.53
High	754	186 ± 31.3	176 ± 16.1	186 ± 33.3	117 ± 19.7	131 ± 26.9	123 ± 13.1	138 ± 23.6	107 ± 18.1	121 ± 23.4	77.8 ± 14.9	98.4 ± 19.6	83.5 ± 8.82	103 ± 27.8	51.2 ± 12.4	77.6 ± 10.6
**Race/Ethnicity**																
White	2479	178 ± 15.5	167 ± 8.2	184 ± 14.2	126 ± 13.3	109 ± 12.5	155 ± 7.93	106 ± 9.49	120 ± 7.62	97.2 ± 9.34	91.2 ± 7.71	104 ± 13.2	82.4 ± 5.58	92.4 ± 10.2	53.0 ± 5.98	73.7 ± 5.28
Black	1646	166 ± 16.3	120 ± 7.0	129 ± 16.7	88.2 ± 10.2	110 ± 15.9	154 ± 8.22	254 ± 17.3	86.9 ± 7.60	113 ± 13.1	95.1 ± 9.92	46.2 ± 8.02	88.6 ± 8.30	87.6 ± 6.53	84.4 ± 10.4	74.9 ± 5.38
Hispanic	1627	193 ± 21.3	131 ± 8.3	105 ± 12.6	212 ± 19.1	314 ± 27.5	108 ± 7.23	147 ± 16.7	86.4 ± 7.41	73.4 ± 10.2	74.0 ± 7.75	40.4 ± 6.68	73.1 ± 5.74	55.3 ± 8.50	33.7 ± 5.03	59.8 ± 4.53
Asian	853	139 ± 20.7	216 ± 16.0	75.2 ± 15.9	319 ± 33.3	81.3 ± 17.2	72.8 ± 6.41	163 ± 21.6	42.1 ± 6.20	55.9 ± 8.91	37.3 ± 10.7	66.7 ± 12.2	71.2 ± 6.37	36.3 ± 7.03	31.2 ± 8.97	114 ± 9.30
“Other”	476	197 ± 38.4	164 ± 24.6	142 ± 39.9	115 ± 24.1	136 ± 33.7	128 ± 17.3	154 ± 31.9	112 ± 19.2	199 ± 50.0	86.0 ± 19.9	99.5 ± 21.4	109 ± 24.0	69.5 ± 18.2	57.8 ± 11.6	69.1 ± 12.5
**Dietary Reference Intakes Age–Sex Groupings**																
>1 y	299	103 ± 23.4	72.0 ± 9.72	51.2 ± 11.9	56.6 ± 12.3	74.6 ± 32.3	140 ± 17.3	53.4 ± 9.42	102 ± 13.8	54.3 ± 12.4	18.6 ± 6.07	24.0 ± 8.39	56.5 ± 8.30	82.5 ± 18.7	99.1 ± 16.5	33.4 ± 5.87
4–8 y	623	180 ± 23.3	114 ± 9.25	126 ± 22.1	72.3 ± 12.4	76.2 ± 17.5	162 ± 13.0	83.6 ± 10.4	98.0 ± 9.49	74.4 ± 11.4	49.2 ± 8.66	40.3 ± 12.9	93.2 ± 8.79	54.6 ± 8.84	86.1 ± 12.0	30.9 ± 3.66
9–13 y males	328	300 ± 52.7	140 ± 14.9	115 ± 22.0	70.3 ± 15.0	199 ± 50.4	271 ± 39.1	111 ± 30.9	86.0 ± 15.3	96.0 ± 26.5	58.2 ± 14.6	29.8 ± 10.4	82.3 ± 10.9	78.2 ± 14.3	89.0 ± 25.0	29.4 ± 6.02
9–13 y females	353	257 ± 45.0	116 ± 14.8	88.2 ± 19.9	107 ± 22.7	60.7 ± 14.1	185 ± 23.8	103 ± 20.5	93.9 ± 15.9	111 ± 23.9	45.4 ± 10.1	51.6 ± 16.8	106 ± 12.9	42.4 ± 9.57	83.5 ± 19.3	40.7 ± 6.44
14–18 y males	341	369 ± 62.6	175 ± 32.3	248 ± 66.2	154 ± 40.7	143 ± 34.5	205 ± 25.1	184 ± 48.3	92.7 ± 18.2	119 ± 26.5	157 ± 25.8	50.0 ± 18.0	79.5 ± 17.8	53.8 ± 14.4	119 ± 23.8	25.6 ± 5.34
14–18 y females	323	206 ± 39.8	148 ± 21.0	106 ± 30.1	99.8 ± 19.8	115 ± 34.3	169 ± 23.5	207 ± 39.1	77.7 ± 15.1	74.4 ± 19.1	58.9 ± 12.7	28.3 ± 12.6	75.0 ± 12.9	35.1 ± 10.4	87.8 ± 24.5	43.0 ± 8.08
19–30 y males	422	295 ± 52.8	134 ± 16.7	254 ± 50.1	73.6 ± 21.5	314 ± 49.1	137 ± 16.0	172 ± 34.9	87.8 ± 12.8	118 ± 25.5	145 ± 22.3	101 ± 23.2	29.9 ± 4.96	84.8 ± 18.8	101 ± 26.5	55.7 ± 9.65
19–30 y females	436	175 ± 31.6	110 ± 12.7	86.6 ± 19.1	99.6 ± 23.8	186 ± 36.4	129 ± 17.6	131.4 ± 17.4	111 ± 15.3	116 ± 24.3	65.8 ± 12.8	88.4 ± 18.3	58.9 ± 11.3	93.0 ± 39.1	73.1 ± 16.2	86.9 ± 10.0
31–50 y males	677	253 ± 37.7	187 ± 19.9	173 ± 31.4	226 ± 40.8	273 ± 43.0	130 ± 14.6	230 ± 32.0	129 ± 21.4	88.7 ± 22.6	150 ± 21.9	51.0 ± 9.57	89.5 ± 14.6	108 ± 19.7	36.7 ± 12.2	78.8 ± 10.7
31–50 y females	744	145 ± 27.2	148 ± 17.4	106 ± 17.2	138 ± 20.8	153 ± 29.0	122 ± 15.8	123 ± 15.9	102 ± 9.89	72.3 ± 11.9	68.3 ± 13.6	60.6 ± 19.4	76.9 ± 10.8	93.6 ± 30.4	29.8 ± 6.78	106 ± 15.3
51–70 y males	876	151 ± 35.2	215 ± 17.9	233 ± 37.3	170 ± 28.5	114 ± 22.5	160 ± 20.8	149 ± 21.4	132 ± 19.1	144 ± 30.8	98.3 ± 16.0	117 ± 32.4	114 ± 15.3	99.1 ± 14.1	20.5 ± 6.46	79.6 ± 10.5
51–70 y females	900	91.3 ± 24.4	160 ± 17.4	112 ± 16.5	178 ± 26.9	61.6 ± 12.4	103 ± 9.94	94.6 ± 12.6	101 ± 15.5	91.7 ± 17.1	65.5 ± 14.0	131 ± 38.1	73.5 ± 12.2	51.8 ± 8.08	26.6 ± 7.63	89.9 ± 10.4
>70 y males	390	84.5 ± 33.8	204 ± 19.6	262 ± 57.1	243 ± 54.6	29.3 ± 9.52	87.8 ± 11.0	86.2 ± 21.3	71.0 ± 10.1	82.1 ± 25.5	68.3 ± 18.9	163 ± 45.0	132 ± 14.5	102 ± 24.5	28.9 ± 11.5	72.9 ± 10.3
>70 y females	369	29.0 ± 9.98	172 ± 14.9	152 ± 27.1	191 ± 40.8	42.1 ± 16.6	126 ± 19.3	85.6 ± 16.1	107 ± 15.5	94.0 ± 21.9	32.6 ± 9.63	110 ± 28.2	99.0 ± 11.9	79.4 ± 18.9	17.0 ± 6.71	104 ± 10.9

^1^ Means ± SE were generated using a PROC SURVEYMEANS procedure in SAS 9.4. All estimates were generated from one 24 h dietary recall and survey weighted to be representative at the population level. Standard errors were computed using the Taylor series linearization method to account for the complex survey design of NHANES. ^2^ This analysis used 87 food categories, which were adapted from What We Eat In America (https://www.cdc.gov/nchs/nhanes/wweia.htm) (accessed on 5 December 2020). Food categories are ranked in descending order by population proportion among the total sample aged >1 y, excluding pregnant and lactating women (*n* = 7081). ^3^ Poverty-to-income ratio (PIR) was used to define household income. PIR ≤ 180% was considered “low” income, 180 < PIR ≤ 350 was considered “middle” income and PIR > 350 was considered “high” income.

## Data Availability

Data are publicly and freely available without restriction at NHANES; https://www.cdc.gov/nchs/nhanes/index.htm, accessed on 20 November 2020.

## Supplementary Materials

The following supporting information can be downloaded at: https://www.mdpi.com/article/10.3390/nu15040831/s1, Table S1. Summary of Food Categories, modified from “Sources of Sodium Potassium Supplement”. Retrieved from [16] and contribution to total daily sodium by food category (%), NHANES 2017-2018 for total sample (*n* = 7081).
