# Supplementary material for: Top Sodium Food Sources in the American Diet—Using National Health and Nutrition Examination Survey
Source: Nutrients. 2023 Feb 6;15(4):831. doi: 10.3390/nu15040831 (PMC9962803; doi:10.3390/nu15040831)
Supplement: Supplementary file 1 [file nutrients-15-00831-s001.zip › nutrients-2081167-supplementary.pdf]

nutrients

MDPI

Supplementary Material

Supplementary Table S1. Summary of Food Categories, modified from "Sources of Sodium Potassium Supplement". Retrieved from https://stacks.cdc.gov/view/cdc/91457 and contribution to total daily sodium by food category (%), NHANES 2017-2018 for total sample (n=7081)

Food Category and Description	% Contribution to daily sodium
Pizza	5.3%
Breads, rolls and buns	4.7%
Cold cuts and cured meats (excluding sandwiches)	4.6%
Soups	4.4%
Burritos and tacos	4.3%
Savory snacks (e.g., chips, crackers, popcorn)	4.1%
Poultry (excluding nuggets and tenders)	4.0%
Cheese (including cottage and ricotta)	3.1%
Pasta mixed dishes, excludes macaroni and cheese	2.9%
Burgers	2.5%
Meat mixed dishes	2.5%
Cookies, brownies, cakes	2.4%
Bacon, frankfurters, sausages (excluding sandwiches)	2.4%
Vegetables (excluding white potatoes)	2.2%
Tomato-based condiments	1.9%
Poultry mixed dishes	1.9%
Mashed, baked or boiled white potatoes	1.9%
Eggs and omelets	1.8%
Salad dressings and vegetable oils	1.7%
Beef	1.6%
Rice	1.5%
Chicken nuggets and tenders	1.5%
Poultry sandwiches	1.5%
Ready to eat cereals	1.4%
Fried white potatoes	1.4%
Hot dogs and sausage sandwiches	1.3%

Dips, gravies, other sauces	1.3%
Quesadillas, tamales, fajitas, enchiladas	1.2%
Fish	1.1%
Biscuits, muffins, quick breads	1.1%
Milk, unflavoured	1.1%
Non-meat sandwiches	1.1%
Stir-fry and soy-based sauce mixtures	1.1%
Other meat (including seafood) sandwiches	1.0%
Tap and bottled water	1.0%
Beans, peas, legumes	1.0%
Other meat (e.g., pork, lamb, other)	1.0%
Pancakes, waffles, French toast	1.0%
Soft drinks, fruit drinks and sport/energy drink	1.0%
Fried rice and lo/chow mein	0.9%
Doughnuts, sweet rolls, pastries	0.9%
Egg sandwiches	0.8%
Rice mixed dishes	0.7%
Macaroni and cheese	0.7%
Soy-based condiments	0.7%
Other condiments	0.7%
Olives, pickles, pickled vegetables	0.6%
Shell fish	0.6%
Tortillas	0.6%
Ice cream, milk shakes and frozen dairy desserts	0.6%
Nuts and seeds	0.6%
Turnovers and other grain-based items	0.6%
Cooked cereals	0.5%
Egg rolls, dumplings and sushi	0.5%
Mayonnaise	0.5%
Seafood mixed dishes	0.5%
Vegetable mixed dishes	0.4%
Not included in a food category	0.4%
Butter, margarine, and animal fats	0.4%
Alcoholic beverages	0.4%
Nutritional beverages and powders	0.4%

Milk, flavoured	0.3%
Coffee	0.3%
Diet beverages	0.3%
Candy	0.3%
Cereal and nutrition bars	0.3%
Bean, pea, legume dishes	0.3%
Nachos	0.3%
Pasta sauces, tomato-based	0.2%
Pasta, noodles, cooked grains	0.2%
Tea	0.2%
Yogurt	0.2%
Milk substitutes	0.2%
Cream and cream substitutes	0.1%
Vegetable juice	0.1%
Flavoured, carbonated and enhanced water	0.1%
Smoothies and grain drinks	0.1%
Pudding	0.1%
Sugars, honeys and other sweeteners	0.1%
Cream cheese, sour cream, whipped cream	0.1%
Fruit	0.1%
100% fruit juice	0.1%
Processed soy products	0.1%
Gelatins, ices, sorbets	0.0%
Baby foods and beverages	0.0%
Infant formula	0.0%
Human milk	0.0%
